# Geographic differences in maternal and child health care utilization in four Ethiopian regions; a cross-sectional study

**DOI:** 10.1186/s12939-019-1079-y

**Published:** 2019-11-12

**Authors:** Atkure Defar, Yemisrach B. Okwaraji, Zemene Tigabu, Lars Åke Persson, Kassahun Alemu

**Affiliations:** 1 Ethiopian Public Health Institute, Addis Ababa, Ethiopia; 2 Department of Epidemiology and Biostatistics, Institute of Public Health, College of Medicine and Health Sciences, University of Gondar, Gondar, Ethiopia; 3 London School of Hygiene & Tropical Medicine, London, UK; 4 Department of Paediatrics and Child Health, School of Medicine and Health Science, University of Gondar, Gondar, Ethiopia

**Keywords:** Ethiopia, Utilization, Antenatal care, Immunization, Childhood illness, Spatial analysis, Autocorrelation, Geographical variation, Ordinary Least Square, Getis Ord Gi*

## Abstract

**Background:**

Maternal and child health (MCH) care utilization often vary with geographic location. We analyzed the geographic distribution and determinants of utilization of four or more antenatal care visits, health facility delivery, child immunization, and care utilization for common childhood illnesses across four Ethiopian regions.

**Methods:**

A cross-sectional community-based study was employed with two-staged stratified cluster sampling in 46 districts of Ethiopia. A total of 6321 women (13–49 years) and 3110 children below the age of 5 years residing in 5714 households were included. We performed a cluster analysis of the selected MCH care utilization using spatial autocorrelation. We identified district-specific relationships between care coverage and selected factors using geocoded district-level data and ordinary least squares and hotspot analysis using Getis Ord Gi*.

**Results:**

Of the 6321women included in the study, 714 had a live birth in the 12 months before the survey. One-third of the women (30, 95% CI 26–34) had made four or more antenatal visits and almost half of the women (47, 95% CI 43–51) had delivered their most recent child at a health facility. Nearly half of the children (48, 95% CI 40–57) with common childhood illnesses (suspected pneumonia, diarrhoea, or fever) sought care at the health facilities. The proportion of fully immunized children was 41% (95%, CI 37–45). Institutional delivery was clustered at district level (spatial autocorrelation, Moron’s I = 0.217, *P* < 0.01). Full immunization coverage was also spatially clustered (Moron’s I = 0.156, *P*-value < 0.1). Four or more antenatal visits were associated with women’s age and parity, while the clustering of institutional delivery was associated with the number of antenatal care visits. Clustering of full immunization was associated with household members owning a mobile phone.

**Conclusions:**

This study showed evidence for geographic clustering in coverage of health facility deliveries and immunization at the district level, but not in the utilization of antenatal care and utilization of health services for common childhood illnesses. Identifying and improving district-level factors that influenced these outcomes may inform efforts to achieve geographical equitability and universal health coverage.

## Background

Improving the utilization of maternal and child health services has been an important way of reducing the risk of maternal and child deaths. In Ethiopia, efforts have been made to improve the utilization of key maternal and child health services, resulting in an increase in the use of these services in the past decades. For instance, from 2005 to 2015, the utilization of four or more antenatal care visits increased from 12 to 32%, and skilled birth attendance increased from 6 to 28% [1].

Despite these overall improvements, there are geographic variations in the utilization of these services [2, 3]. A further analysis of the Ethiopian 2016 Demographic and Health Survey (DHS) revealed a geographic variation in postnatal care coverage (Moran’s I = 0.08) [4]. Similarly, a study conducted to assess the geographical variation of antenatal care utilization showed higher utilisation in urban settings and lower in pastoralists regions [5].

There is also geographic variation in childhood illness and utilization of child health services across Ethiopia as well as across the African continent [2, 6, 7]. A systematic review conducted to assess the geographical variation of care utilization in 27 selected African countries showed variation in immunization coverage and care-seeking at national and subnational levels [2, 8]. A low coverage of immunization at national and subnational levels was observed to a larger extent in Ethiopia as compared to other countries included in the study. Furthermore, the study reported variation in care-seeking for common childhood illness between countries. It was found to be low in Ethiopia (29%) and high in Uganda (78%). Clustering of low values was mainly shown in western Africa, Ethiopia, Zimbabwe, and a small section of central Africa [2].

The geographical characteristics of an area with variations in travel time to the nearest health facility are found to be the main explanation to the geographical variation in the utilization of maternal and child health services [9]. There are some studies that used geographic information system (GIS) modelling to explore factors associated with the geographical clustering of these services [10, 11]. However, these studies did not explore factors associated with the geographic clustering using advanced spatial methods [12, 13].

There is a range of GIS-based advanced spatial methods that provide appropriate analyses for the assessment of the geographic variation of health care utilization [14–16]. Such methods allow for explicit use of GIS-linked data to study the geographical variation of care utilization [17] and perform in-depth analyses of factors associated with the geographical variation [18, 19].

Thus, the aim of this study was to identify the geographic variation of selected maternal and child health services utilization (antenatal care, institutional delivery, child immunizations, and care-seeking for common childhood illness) and the factors associated with the observed spatial variation in four regions of Ethiopia by applying a geographical analysis approach including spatial pattern analysis global (Spatial Autocorrelation) and Ordinary Least Square method (OLS).

## Methods

### Study setting and population

**Fig. 1:**
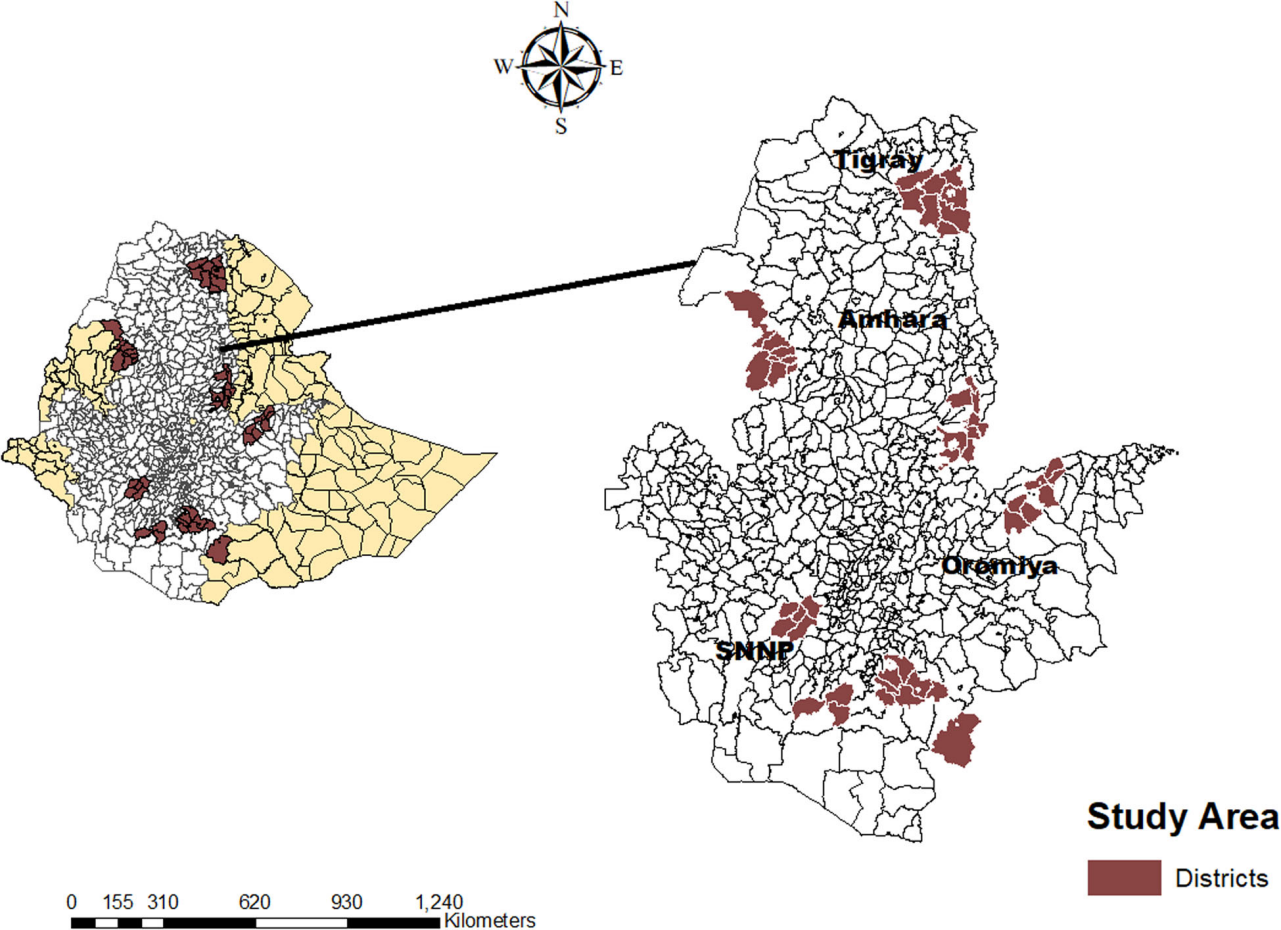
Map of Ethiopia indicating the regions and districts where the study was undertaken

The study was conducted in 46 districts in the four most populous regions of Ethiopia: Tigray, Oromia, Amhara, and the Southern Nations, Nationalities and Peoples (SNNP) (Fig. 1). According to the 2018 UN population estimates, the population size of Ethiopia was 107,099,196 with 79% of the population living in rural settings and over 80% of the country’s population residing in these four regions. The topography is dominated by hilly areas.

### Study design

The study was a cross-sectional community-based survey conducted from December 2016 to February 2017 in intervention and comparison districts selected for the implementation of the Optimizing the Health Extension Program (OHEP), which aimed at increasing the utilization of health services for sick newborns and children below the age of 5 years. The comparison districts had similar socio-demographic characteristics as the intervention districts. The survey was performed prior to the implementation of OHEP. The evaluation of this intervention was registered in the Current Controlled Trials ISRCTN12040912.

### Sampling

A two-stage stratified cluster sampling was applied in the selected study districts. The first stage used lists of enumeration areas (EA) from the 2007 Ethiopian Housing and Population Census as the sampling frame [20]. The cumulative population size of enumeration areas across the study areas was calculated, and 200 EAs were selected with probability proportional to size. Each EA formed one cluster, and these clusters constituted the primary sampling unit.

In the second stage, a systematic random sampling technique was used. All households within each cluster were listed and a sampling interval was calculated. A random start number between one and the sampling interval was selected. The households that matched the random start number in the list was thereafter selected as the first household to be included. This process was repeated until the targeted number of 30 households in each cluster was reached. All women aged 13 to 49 years and children under the age of 5 years, who lived in the selected households, were included in the survey.

We used a standard sample size formula with a design effect of 1.3. We used 80% power and the assumption of a ratio of 0.65 children less than 5 years of age per household, based on sampling reported in the Ethiopia DHS 2011. Finally, a total sample size of 6000 households was estimated.

### Study participants

Women of reproductive age (13–49 years), who had given birth during the last year, and children aged less than 5 years were the primary subjects of this study. Mothers or caregivers of the included children were interviewed regarding their care-seeking behaviour for themselves and their children.

### Survey methods

There were 15 data collection teams; each team comprised one team leader and three enumerators. Data were collected using tablet computers. Data collectors were trained for 10 days covering study procedures, questionnaires, data collection techniques, quality assurance procedures, and study ethics. A field manual was provided to data collectors and the survey tools were pilot-tested in the field during the last 3 days of the training.

The interviewers first obtained informed consent from the house head and collected information on socio-demographic characteristics of the household and all members of the household. This was followed by interviews of all women of reproductive age (13 to 49 years) to get information on live births in the 12 months preceding the survey including information on utilization of antenatal care and delivery care, and their knowledge of pregnancy and newborn danger signs. Childhood immunization status, children’s two-weeks morbidity recalls, and health care utilization for any recent child illness episode were also collected from caregivers or mothers of children between the age of 2–59 months who resided in the household. The questionnaire modules were based on existing large-scale survey tools, such as the Demographic and Health Surveys, the Service Provision Assessment, the Averting Maternal Death and Disability, and Safe Motherhood survey tools and had been piloted and adapted for this survey.

### Outcome variables

We estimated the overall coverage of four selected maternal and child health services. For women, this included the utilization of four or more antenatal care visits and institutional delivery. For children 2–59 months with illness during the last 2 weeks, care-seeking to health posts, health centers, hospitals, or private clinics was included. Further, we assessed the proportion of children aged 12–23 months, who were fully immunized in accordance with the national immunization program (Table 1).

**Table 1:** Definition of the studied maternal and child health services utilization

Health service	Definition
Four or more antenatal care visits	The proportion of women aged 13 to 49 with a live birth in the last 12 months who attended antenatal care four or more times during that pregnancy.
Institutional delivery	The proportion of women with a live birth in the year preceding the survey who gave birth in a health facility
Child immunization; fully immunized	The proportion of live children aged 12–23 months who had received Bacillus Calmette-Guerin (BCG) vaccine, three doses of polio vaccine and three doses of pentavalent vaccine (Diphtheria-Tetanus-Pertussis-Hepatitis B and Hemophilus vaccines)
Care seeking for common childhood illness	The proportion of children aged 2–59 months with fever, diarrhoea, or suspected pneumonia in the last two weeks for whom care was sought at health posts, health centers, hospitals, or private clinics

### Explanatory variables

Selected environmental, household, and individual factors were included in the analytical models and assessed for their random geographical distribution across the study areas before using in the spatial analysis. Variables considered as household factors were having family health cards, owning mobile, radio, or television set in the household, participation in women’s development groups, and the number of pregnancies and births. Variables considered as individual factors were education status of the mother, age of the mother and the child, sex of the child, and the number of health facility visits. Health system-level factors were the availability of health post in the village (kebele) and reported distance to the nearest health post. The altitude of the household was considered an environmental factor.

### Spatial variables

Shapefiles of each district were obtained from the Central Statistical Agency, Ethiopia. These data had the X and Y coordinates and spatial areas of districts, which made up the polygon file for each district in the study areas.

### Data analyses

#### Measuring outcomes and explanatory variables

Descriptive analysis was done to generate service utilization at the district level for selected outcome indicators. These proportions for the use of maternal and child health services were calculated from the total women aged from 13 to 49 years with live births in the 12 months period prior to the study, children from 2 to 59 months old, and children from 12 to 23 months old.

District level mean values of continuous variables and proportions of categorical variables were also calculated for explanatory variables. All analyses were done using STATA 14 software (StataCorp LLC, Texas, United States).

#### Spatial variation of maternal and child health care utilization

A database was created including spatial information, care utilization outcomes, and explanatory variables. The outcome and explanatory variables were linked with spatial data at the district level using ArcGIS Desktop v10.5 (Environmental Systems Research Institute Inc., Redlands CA, USA). The Spatial Autocorrelation matric equation is expressed [21] as;

$$ I(d)=\frac{\frac{1}{W}\;\sum \limits_{k-1}^n\sum \limits_{i-1}^n{W}_{ki}\;\left({y}_k-\overline{y}\right)\;\left({y}_i-\overline{y}\right)}{\frac{1}{n}\;\sum \limits_{i-1}^n{\left({y}_i-\overline{y}\right)}^2} $$

Where: *I(d)* = Moran’s *I* correlation coefficient as a function of distance. W_*ki*_ = a matrix of weighted values, where elements are a function of distance 1 = y_h_ and y_i_ are within a given distance class, for y_h_ y_i_ 0 = all other cases. The value of *I* could depend on the assumptions built into the spatial weight’s matrix W_ij_.

Maternal and child health care utilization data at the district level were assessed for geographical clustering using spatial autocorrelation (Moran’s Index). The spatial autocorrelation analysis estimated the Moran’s Index and the z score, and its associated *p*-values were computed for observed and expected index values, given the number of features and the variance of the data values. A positive Moran’s Index value indicates a tendency toward clustering while a negative Moran’s Index value indicates a tendency toward dispersion. This analytical model hypothesized that the geographical distribution of utilization of the selected maternal and child health services was randomly distributed. The concept implies that nearby locations tend to be more alike than locations that are far away from one another [17]. Thus, the analysis measures spatial autocorrelation based on feature locations and attribute values. It estimates within and across regional and district boundaries using the Moran’s I statistic to decide whether the pattern expressed is clustered, dispersed, or random.

#### Linear regression and hot spot analysis

The global Ordinary Least Square (OLS) linear regression method was used to predict the relationship between selected explanatory variables and the outcome variables. Spatial autocorrelation in the residuals of the OLS models was thereafter again tested by Moran’s I statistics to ascertain that the residual was not clustered. A spatial autocorrelation coefficient, which may range from − 1 (negative autocorrelation) to + 1 (positive spatial autocorrelation), was used to decide. If we found the existence of the spatial autocorrelation in the residuals, the OLS model would not fit the data set and further refined spatial models should be used. In such a case, a hot spot analysis was done using Getis Ord Gi*, which estimates a z-score of the observed and expected spatial clustering with high or low values and its associated *p*-values. The result of Getis Ord Gi* tells us how strong the linear model is within and between geographic areas and identifies which areas that contribute to a high or low degree in the spatial regression modelling. In this process, a value of 1 in the inverse distance conceptualizations of spatial relationships was used to avoid computation of the default threshold and row standardization was applied.

## Results

### Participants’ characteristics

A total of 6321 women aged 13–49 years living in the surveyed 5714 households were interviewed about their recent birth histories. Of these, 714 had a live birth in the last 12 months preceding the survey.

Caregivers of 3110 children under the age of 5 years living in the surveyed households were interviewed. Of these children, 2873 (92%) were aged 2–59 months and 576 (16%) were between 12 and 23 months.

The participating women were from Oromia (53%), Amhara (24%) Southern Nation, Nationality and People (13%) and Tigray regions (10%). More than half (53%) of the women had no education while only 10% had completed secondary school or above. Forty percent of women who had a live birth during the last 12 months preceding the survey were orthodox Christian, followed by Muslims (30%) and protestants (28%).

### Maternal and child health care utilization

**Fig. 2:**
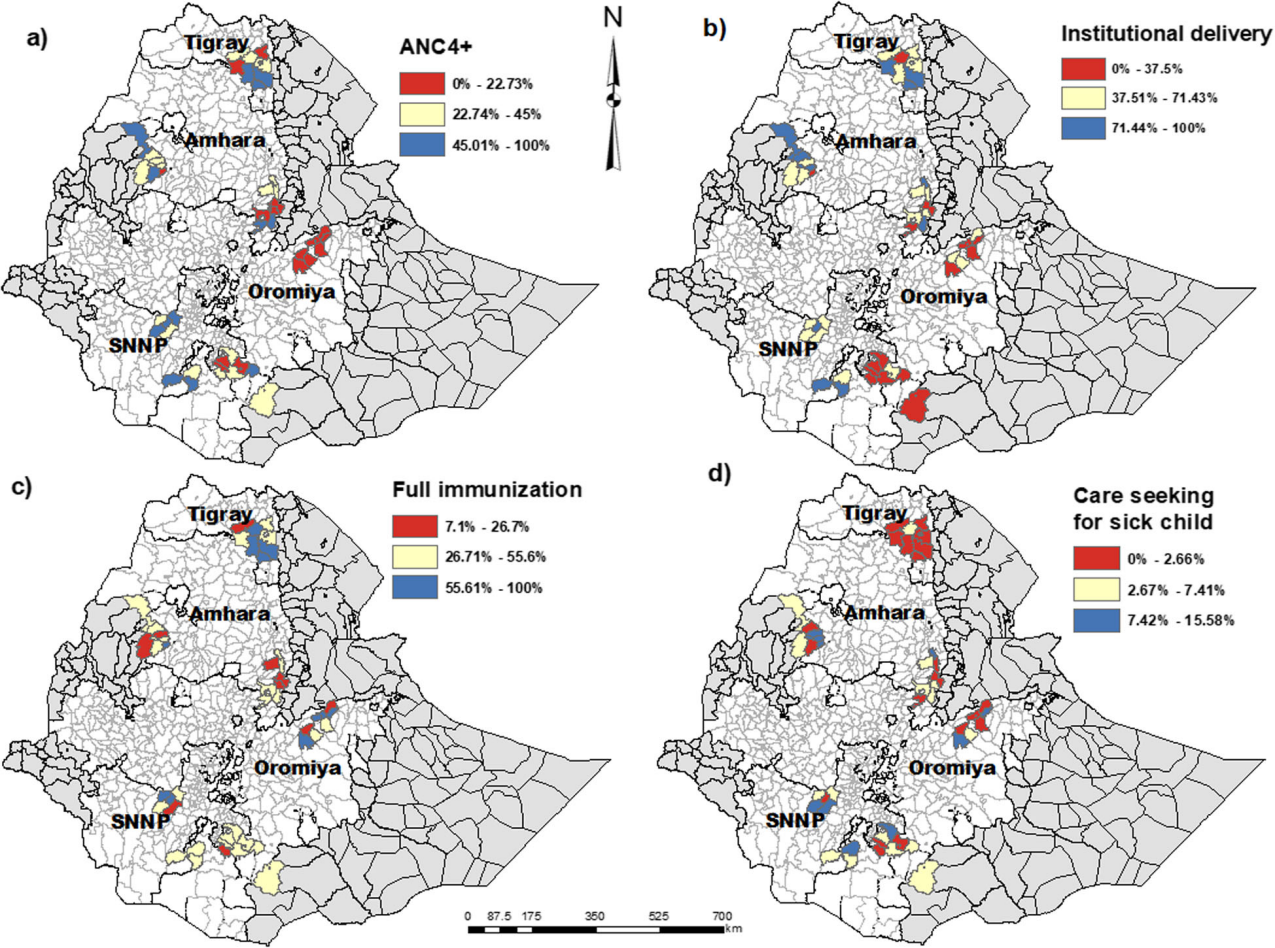
Maps showing geographic distribution of maternal and child health care service use in 46 districts of four regions of Ethiopia. **a** Four or more antenatal care visits **b**) Health facility delivery care **c**) Full immunization **d**) Care seeking for common childhood illness. The three levels of utilization (red, yellow, blue) for each service utilization represent the tertile distributions

Around one-third of the women with birth during the last year had attended antenatal care four or more times and almost half had delivered at a health facility (Table 2 and Fig. 2). The overall prevalence of reported common childhood illnesses (suspected pneumonia, diarrhoea or fever) in the 2 weeks prior to the survey was 139/2873 (5%; 95% CI 3–7). Of these, 48% (95% CI 40–57) sought care from a formal health provider (health post, health center, hospital or private clinic). The proportion of fully immunized children (12–23 months) was 41% (95% CI: 37–45).

**Table 2:** Level of utilization and indications of geographic clustering of use of selected maternal and child health services in four national regions of Ethiopia, December 2016–February 2017

Care utilization indicators	n/N	% (95% CI)	Moran’s Index	Z-score of the Index	*P*-value
Maternal health services					
Four or more antenatal care visits	215/714	30 (27–35)	0.089	1.351	0.176
Delivery in a health facility	336/714	47 (43–51)	0.205	2.70	0.006
Child health services					
Fully immunized children 12–23 months age	232/567	41 (37–45)	0.172	2.30	0.021
Utilization of care for common childhood illnesses during the last 2 weeks	67/139	48 (40–57)	0.016	0.454	0.640

### Spatial patterns in the utilization of maternal and child health care

**Fig. 3:**
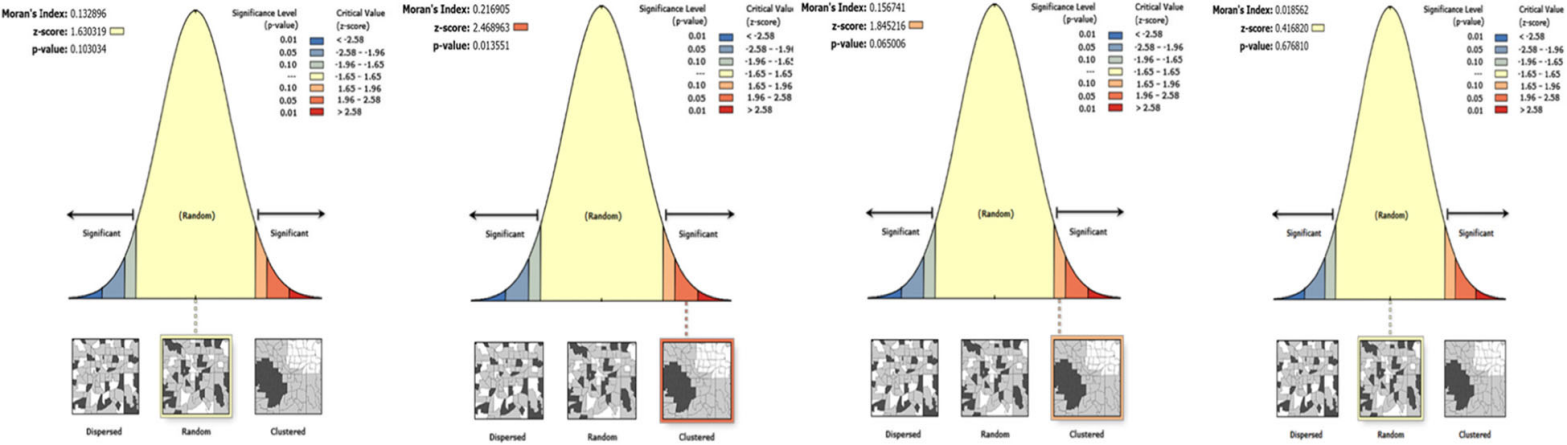
Spatial patterns of maternal and child health care utilization in 46 districts of the four regions of Ethiopia. The clustered patterns (on the right sides) show that similar coverage values at the district level are close to each other. The outputs have automatically generated keys on the right and left sides of each panel. Auto-generated interpretations available below each panel shows whether the likelihood of clustered patterns occurred randomly is less than 1, 5% or 10%. The bright red and blue colours (to the end tails) indicate increased significance levels. Left to right, the graphs show 1) Antenatal care visits four or more times; 2) Health facility delivery care; 3) Full immunization coverage, and 4) Sick child care-seeking for common childhood illnesses

The presence of geographical clustering was analyzed for all selected outcome indicators using global spatial autocorrelation (Moran’s’ Index) as presented in Table 2. There were indications of geographic clustering in the utilization of facility delivery (Moran’s I = 0.205, *p* = 0.006) and full immunization coverage (Moran’s I = 0.172, *p* = 0.021) (Table 2, Figs. 2 and 3). However, there was neither indication of geographic clustering of four or more antenatal care visits nor of the utilization of health care when the under-five child was sick.

### Factors associated with maternal and child health care utilization

The age and parity of women were associated with the utilization of antenatal care visits four or more times, while the number of antenatal care visits was associated with institutional delivery (Table 3). Caregivers’ ownership of mobile phones was associated with full child immunization coverage, while none of the selected socio-demographic or health system characteristics was related to sick child care-seeking.

**Table 3:** Association between selected socio-geographic and health system factors and the geographical distribution of maternal and child health care utilization in 46 districts of Ethiopia, December 2016–February 2017

Explanatory variables^a^	Estimate	Standard error	*t-value*	*p* value	Vif^b^
Antenatal care visits four times or more					
Intercept	0.283				
Mean age of the women	0.013	0.015	2.157	0.037**	1.652
Mean education level of the women	−0.018	0.024	−0.798	0.429	1.672
The proportion of households with a mobile phone	−0.002	0.000	0.911	0.368	1.606
Proportion of households with a radio	0.000	0.000	1.083	0.285	1.799
Proportion of women’s ownership of households	−0.002	0.004	− 0.544	0.589	1.157
Proportion of women having a family health card	0.003	0.002	1.437	0.159	1.240
Mean parity	−0.111	0.039	−2.882	0.006**	1.724
Health facility delivery					
Intercept	−0.195				
Mean age of the women	0.01	0.016	0.591	0.558	2.578
Mean number of pregnancies	−0.106	0.162	− 0.652	0.518	1.812
Mean number of antenatal care visits	0.007	0.002	3.013	0.004**	1.721
The proportion of women participating in women’s development groups	0.001	0.002	0.646	0.522	1.167
Proportion of women having a family health card	0.003	0.002	1.484	0.145	1.196
Mean parity	−0.006	0.055	−0.121	0.904	4.207
Full immunization					
Intercept	0.309				
Mean distance to the health post	−0.001	0.001	− 0.91	0.368	1.406
Family size	−0.05	0.048	−1.05	0.299	1.515
Mean radio availability in the household	−0.17	0.172	−0.987	0.330	1.154
Mean mobile phone availability in the household	0.206	0.109	1.896	0.046**	1.274
Mean elevation	−0.000	0.000	−0.65	0.519	1.273
Mean age of the child (in month)	0.046	0.031	1.498	0.143	1.209
The proportion of male children	−0.195	0.225	−0.867	0.392	1.705
Proportion of household with health post in their kebele	0.179	0.229	0.785	0.437	1.265
Sick child care utilization					
Intercept	−0.221				
The proportion of household with health post in their kebele	0.007	0.005	1.337	0.189	1.15
The proportion of male children	0.004	0.008	0.530	0.599	1.153
Mean distance to health post (reported)	−0.004	0.004	−1.212	0.232	1.055

** Significance at *p-value < 0.05*

^a^The estimation was done at district level

^b^Variance inflation factor

**Fig. 4:**
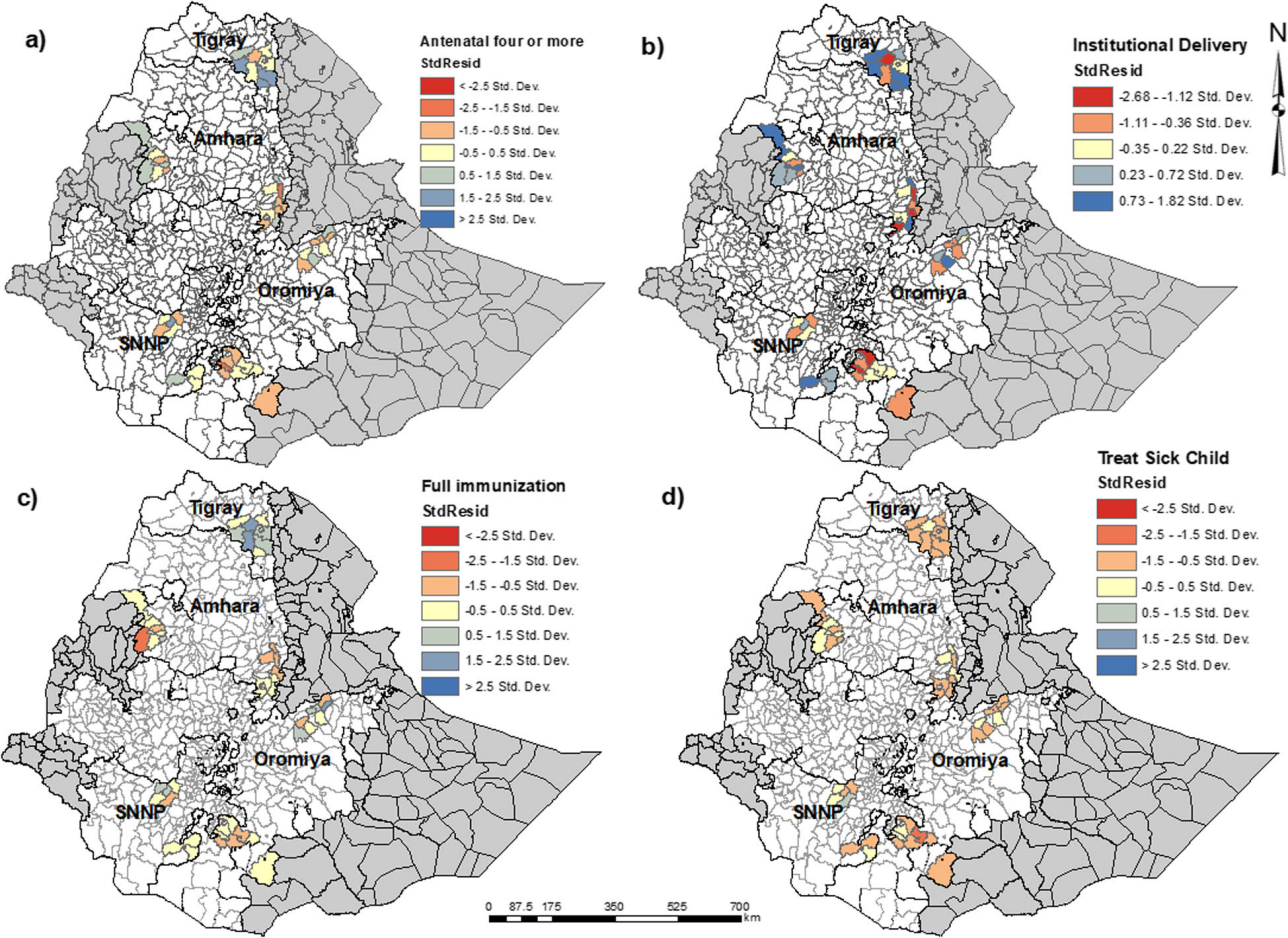
Maps showing the distribution of the standard residuals from the OLS model **a**) Antenatal care visits four times or more **b**) Health facility delivery care **c**) Full immunization **d**) Sick child care-seeking for common childhood illnesses

The distribution of standardized residuals from the Ordinary Least Squares models is shown in Fig. 4.

There was no evidence of spatial clustering of the residual in the OLS model for antenatal care four or more times, health facility delivery, or care-seeking of the sick child. The OLS model residual for full immunization coverage showed a significant spatial clustering (Moran’s I = 0.182 and *p*-value = 0.032) (Table 4). Thus, we ran a Hot Spot Analysis using Getis Ord Gi*.

**Table 4:** Analysis of geographic clustering of residuals in the linear regression analyses of determinants of selected maternal and child health care utilization. Moran’s I test statistics

Outcome indicators	Morans’ Index	Z-score of the Index	*p*-value	Interpretation
Antenatal care visits four times or more	0.025	0.488	0.625	OLS residual was not clustered
Health facility delivery	0.423	0.800	0.423	OLS residual was not clustered
Immunization	0.182	2.136	0.032	OLS residual was clustered
Sick child care utilization	−0.052	− 0.039	0.752	OLS residual was not clustered

### Hot spot analysis

**Fig. 5:**
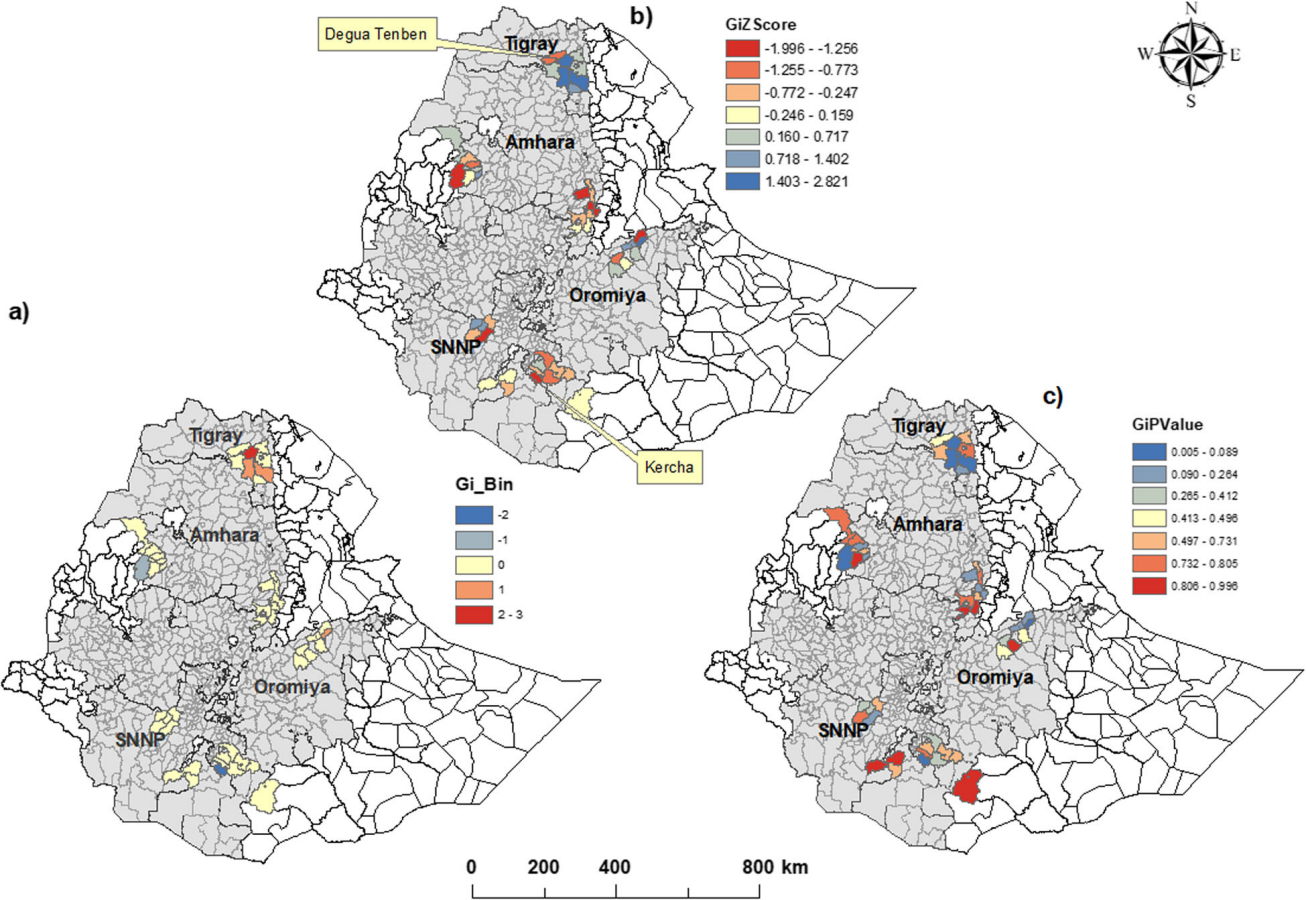
Spatial clustering of high (Hot spot), and low values (cold spot) for full immunization coverage. **a**) The observed +/− 3 Gi_bins in the map reflect statistical significance with 99% confidence level; the +/− 2 Gi_bins reflect a 95% confidence level; the +/− 1 Gi_bins reflect a 90% confidence level; and the clustering for features in bin 0 is not statistically significant. **b** & **c**) The z-score are used to measures the statistical significance given the number of features and their values. It helps to conclude whether to reject or not the null hypothesis, feature by feature

As shown in Fig. 5, some of the districts in Tigray region showed indications of clustering of high levels of full immunization coverage that was not explained by the determinants included in the regression model. In contrast, in Oromia region there were districts with indications of clustered low coverage that was not explained by the included determinants. Other factors, not included in our models, might be associated with the geographical clustering of full immunization.

## Discussion

We have shown that in the selected study districts of four Ethiopian regions there was geographical clustering in the utilization of facility-based delivery and in the coverage of full child immunization. The utilization of four or more antenatal care visits and care-seeking for sick children were, however, randomly distributed in the study districts.

Previous analyses of three consecutive Ethiopian Demographic and Health Surveys showed a regional variation in the attendance to antenatal care [22]. Our study considered use of antenatal care related to births within 12 months prior to the study and applied spatial analysis, whereas the analyses of the Demographic Health Surveys reflected earlier 5-year periods and used conventional statistics to describe the geographical variation.

This study, as well as previous researches, have shown that mothers’ age and parity were positively related to four or more antenatal care visits [23, 24]. Possible reasons for this finding may include that older and higher parity women have experiences that make them more aware of the potential benefits from antenatal care.

Antenatal care may be considered as a motivation for facility-based delivery care [25]. The clustering of facility delivery was associated with the number of antenatal care visits, which is in line with previous studies in Ethiopia [23, 26].

Full immunization coverage was clustered at district level, which indicates that similar levels of immunization coverage (higher or lower) were found in close geographical areas. A study conducted across different African countries found regional level clustering of low or high immunization coverage of measles, pentavalent, and BCG vaccines [27].

When analyzing potential factors that may affect clustering, we could only demonstrate an association between household ownership of a mobile phone and full immunization coverage. Characteristics such as distance to the health post or availability of a health facility in the village were not associated with full immunization. In contrast, researches from other countries have reported that clustering of vaccination was associated with location and distance to health facilities [28–30]. In Ethiopia, however, immunization is to a large extent provided through outreach activities performed by the health extension workers that could explain this difference in findings. The statistical analysis of residuals revealed, however, that we were not able to satisfactorily identify determinants of clustering of full immunization at district level.

In this study, there was no geographical variation in care-seeking for common childhood illnesses and none of the selected sociodemographic and health facility characteristics was found to be associated with care-seeking for the sick child. Other studies have, however, found that availability of health facility in the village, distance to the nearest health facility, and other socio-demographic characteristics to be associated with the geographical variation of care-seeking for the sick child [31, 32].

The current study has strengths and limitations. We selected a range of key maternal and child health services to study the geographical distribution of health service utilization at district level. In addition, we used advanced spatial analysis techniques to identify clustering of care utilization and to identify associated factors. The geographical location of the districts and the relatively small number of data points in our analysis may affect the spatial modelling and we were not able to conduct geographically weighted regression to analyze local-level variation due to the limited number of data points. Instead, we performed ordinary least square analysis to identify associations between individual, socio-demographic, and health services characteristics and care utilization outcomes. The number of sick under-five children was small, which limited the ability to identify geographical clustering of care utilization in the study areas. Other Ethiopian studies have reported geographical clustering of care-seeking for common childhood illnesses as well as geographical clustering of childhood morbidity [33, 34], which also have been found in other sub-Saharan African countries [7, 35, 36].

## Conclusion

This study shed light on the geographical variation in utilization of health facility delivery care and child immunization across four Ethiopian regions [37]. Our finding showed a geographical variability in the use of some maternal and child health services at districts level, suggesting that there are area-specific factors that influence the utilization and coverage of these services. Identifying district-level factors that influence these outcomes may inform efforts to achieve geographical equitability and universal coverage of maternal and child health services in Ethiopia.

## Data Availability

The datasets analyzed in the current study available at the data repository centre of the EPHI. The corresponding author may be contacted for data request.

## Abbreviations

ANC: Antenatal Care
AOR: Adjusted Odds Ratio
CoC: Continuum of Care
COR: Crude Odds Ratio
CSV: Comma Separated values
EDHS: Ethiopian Demographic and Health Survey
EPHI: Ethiopian Public Health Institute
GWR: Geographically Weighted Regression
HF: Health Facility
HH: Household
LSHTM: London School of Hygiene and Tropical Medicine
MNC: Maternal-newborn and Child
MNCH: Maternal-newborn and Child
PNC: Postnatal Care
SA: Spatial Autocorrelation
SA: Spatial Autocorrelation
SERO: Scientific Ethical Review Office
